# Surgical Wards

**Published:** 1841-12-04

**Authors:** E. Peace


### CLINICAL REPORTS.

### PENNYSLVANIA HOSPITAL.

*Surgical Wards, Service of Dr. E. Peace.*

Discharged, Oct. 30, 1841, Ann P—, æt. 27, of a plethoric, scrofulous habit, and sanguine, lymphatic temperament, married, and five months gone with her second child, having miscarried seven months previously, of the first. Admitted Sept. 27, complaining of great pain in the left side of the head, and in left ear, accompanied by a constant, free, and very fetid muco-purulent discharge from the meatus of the latter. Four years ago, last spring, she struck her ear in a fall upon a brick pavement with so much violence as to excite bleeding from the part and cause severe pain of several hours' duration. More or less pain and ringing in the ear continued from that time with little intermission, the pain gradually increasing, until, at the end of six months, it became very acute. After suffering four or five months longer in this manner, without any remedial essay, other than an occasional vesication over the mastoid region of the affected side, she entered the Pennsylvania Hospital. While here, she was cupped repeatedly, blistered at the back of the neck, wore a seton in the neck for two months, and in six months left the institution entirely well.

An interval of two years and three months elapsed without any return of the former symptoms. Early last winter she was attacked with frontal pains darting across from temple to temple, and intense pain in the left ear and side of the head, always more violent towards evening and at night, and much aggravated by the slightest motion, stooping, or exposure to heat. At the same time there appeared from the diseased organ a constant watery discharge which, by degrees, became purulent, and, on the approach of the mild weather of spring, began to be extremely offensive. This state of things continued throughout the summer, until she entered this hospital the second time on the 27th of September.

Specular examination disclosed an apparently superficial ulcer on the membrane of the tympanum, about three lines in diameter, together with redness and an excoriated appearance of that membrane, and the meatus externus generally; there being, moreover, much sensibility of these parts upon the introduction of the instrument. The tonsils and fauces also appeared to be inflamed, and the hearing on that side was so obtuse as to render inaudible the ticking of a watch closely applied to her ear.

She was purged, restricted to a vegetable diet, and cupped freely on the temples and behind the ears. The auditory tube was thoroughly syringed out with tepid water and castile soap, and filled with an injection of creosote water, in the proportion of four drops to the ounce, twice daily, for ten days. No material benefit having been derived from this plan of treatment, further than a temporary correction of the fetor after each dressing, another course was instituted. After a thorough washing of the meatus, as before, burnt alum in powder was carefully thrown into it once a day by insufflation through a quill. The pain abated, and the discharge diminished, while the fetor was almost entirely removed after the second application of this remedy. In thirteen days, all unpleasant symptoms, except the inflammation of the fauces, and some dulness of hearing, had entirely disappeared.

Creosote water (four drops to the ounce) was freely applied to the inflamed surface with a camel's hair pencil once daily for seven days. She was then discharged cured, having recovered her hearing so as to perceive the ticking of a watch at some distance from her ear.

In the above case no perforation of the tympanum, such as might have been expected, was discovered. The examination, however, was not perhaps as complete as it might have been, since the catheterism of the Eustachian tube was not resorted to, although obstruction of this tube was suspected at the time.

*Remarks.*—Burnt alum is an application much in vogue in these cases among the practitioners of Paris. We are indebted for its prescription in the present instance, and its introduction into the practice of this hospital, to Dr. E. Lang, of this city, who has seen it repeatedly employed with great success by the celebrated Deleau.

The solution of the acetate of lead, so strongly recommended by Kramer, and which is really so valuable, especially in correcting the excessive fetor, was not directed for this individual. In relation, however, to the promptness and efficiency of the acetate of lead in removing all unpleasant odour, it may not be out of place to remark that this chemical characteristic alone would render it an extremely useful auxiliary in the dressings of parts affected with fetid discharges.

Attention has been incidentally called to this property of the acetate of lead by Ricord, in his work on the venereal disease, when recommending its employment for certain offensive discharges from the vagina.

The explanation given is that the sulphuretted hydrogen (the evil spirit in most vile smells of organic origin) is decomposed by the lead, and a sulphuret of the latter is precipitated, which becomes evident by blackening the dressings. This antiseptic plan has been tried repeatedly in these wards during the past five months, and always with results so satisfactory that we should be inclined to prefer it—wherever not contra-indicated—to any preparation of chlorine or creosote; both of which are, in themselves, objectionable, because offensive.

